# Clinical and Histologic Variants of CD8+ Cutaneous T-Cell Lymphomas

**DOI:** 10.3390/cancers16173087

**Published:** 2024-09-05

**Authors:** Madisen A. Swallow, Goran Micevic, Amanda Zhou, Kacie R. Carlson, Francine M. Foss, Michael Girardi

**Affiliations:** 1 Yale School of Medicine, New Haven, CT 06510, USA; madisen.swallow@yale.edu; 2 Department of Dermatology, Yale School of Medicine, New Haven, CT 06510, USA; goran.micevic@yale.edu (G.M.); amanda.zhou@yale.edu (A.Z.); kacie.carlson@yale.edu (K.R.C.); 3 Hematology and Stem Cell Transplantation, Yale School of Medicine, New Haven, CT 06510, USA; francine.foss@yale.edu

**Keywords:** cutaneous T-cell lymphoma, CD8+ cytotoxic T-cell lymphoma, CD8+ mycosis fungoides, CD8+ lymphomatoid papulosis, subcutaneous panniculitis-like T cell lymphoma, primary cutaneous gamma/delta T-cell lymphoma, CD8+ AECTCL, acral CD8+ T-cell lymphoproliferative disorder

## Abstract

**Simple Summary:**

CD8+ CTCL subtypes manifest with widespread clinical, histologic, and phenotypic features that inform the classification of the disease. Through this review, we highlight the importance of utilizing the synergy of clinical, histologic, and immunohistochemical findings to determine a correct diagnosis and applicable treatment plan.

**Abstract:**

Although the vast majority of CTCL subtypes are of the CD4+ T-helper cell differentiation phenotype, there is a spectrum of CD8+ variants that manifest wide-ranging clinical, histologic, and phenotypic features that inform the classification of the disease. CD8, like CD4, and cytotoxic molecules (including TIA and granzyme) are readily detectable via IHC staining of tissue and, when expressed on the phenotypically abnormal T-cell population, can help distinguish specific CTCL subtypes. Nonetheless, given that the histopathologic differential for CD8+ lymphoproliferative disorders and lymphomas may range from very indolent lymphomatoid papulosis (LyP) to aggressive entities like CD8+ aggressive epidermotropic cytotoxic T-cell lymphoma (AECTCL), CD8 and/or cytotoxic molecule expression alone is insufficient for diagnosis and is not in itself an indicator of prognosis. We present a review of CTCL subtypes that can demonstrate CD8 positivity: CD8+ mycosis fungoides (MF), LyP type D, subcutaneous panniculitis-like T-cell lymphoma (SPTCL), primary cutaneous gamma/delta T-cell lymphoma (PCGDTL), CD8+ AECTCL, and acral CD8+ T-cell lymphoproliferative disorder (acral CD8+ TCLPD). These diseases may have different clinical manifestations and distinctive treatment algorithms. Due to the rare nature of these diseases, it is imperative to integrate clinical, histologic, and immunohistochemical findings to determine an accurate diagnosis and an appropriate treatment plan.

## 1. Introduction

The term cutaneous T-cell lymphoma (CTCL) encompasses variants of non-Hodgkin’s lymphomas that primarily affect the skin and can involve lymph nodes (LNs) and peripheral blood in more advanced stages [1]. Although the vast majority of CTCL subtypes are of the CD4+ T-helper cell differentiation phenotype, there is a wide range of clinical, histologic, and phenotypic variants. The fifth edition of the World Health Organization Classification of Hematolymphoid Tumours describes primary cutaneous T-cell lymphoid proliferations and lymphomas as a dedicated family with nine underlying entities (Table 1): mycosis fungoides (MF), including Sézary syndrome (SS); primary cutaneous CD30-positive T-cell lymphoproliferative disorder (CD30+ LPD), including lymphomatoid papulosis (LyP); primary CD30+ LPD, including primary cutaneous anaplastic large cell lymphoma (PCALCL); subcutaneous panniculitis-like T-cell lymphoma (SPTCL); primary cutaneous gamma/delta T-cell lymphoma (PCGDTL); CD8-positive aggressive epidermotropic cytotoxic T-cell lymphoma (CD8+ AECTCL); CD8-positive acral T-cell lymphoproliferative disorder (acral CD8+ TCLPD); CD4-positive small or medium T-cell lymphoproliferative disorder (CD4+ SMCLPD); and primary cutaneous peripheral T-cell lymphoma not otherwise specified (PTCL-NOS) [2]. The most recent edition assigns specific designation to PCGDTL, CD8+ AECTCL, acral CD8+ TCLPD, and CD4+ SMCLP, which were previously grouped under the term “cutaneous peripheral T-cell lymphoma, rare types” [2].

**Figure 1:**
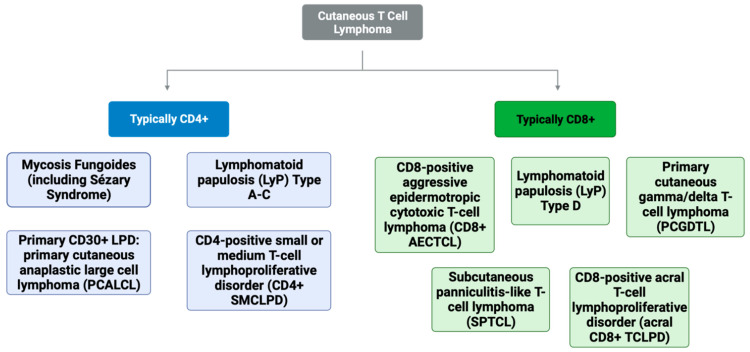
The cutaneous T-cell lymphoma entities divided into typically CD4-positive and CD8-positive lymphomas.

In addition to distinguishing clinical and histologic features, the immunohistochemistry (IHC) of T-cell marker expression can help differentiate these entities. CD8 is a T-cell transmembrane glycoprotein that specifically binds major histocompatibility complex class I, thus serving as a co-receptor for the T-cell receptor recognition of MHC-I presented peptide antigens. CD8, like CD4, is readily detectable via IHC staining of tissue. However, given that the histopathologic differential for CD8+ lymphoproliferative disorders and lymphomas may range from indolent LyP to aggressive entities like CD8+ AECTCL, CD8 expression alone is insufficient for diagnosis and is not in itself an indicator of prognosis. The purpose of this review is to describe the various entities typically demonstrating CD8-positivity, namely CD8+ MF, CD8+ LyP, SPTCL, PCGDTL, CD8+ AECTCL, and acral CD8+ T-cell lymphomas, with respect to clinical features prognoses and treatment regimens (Figure 1).

**Figure 2:**
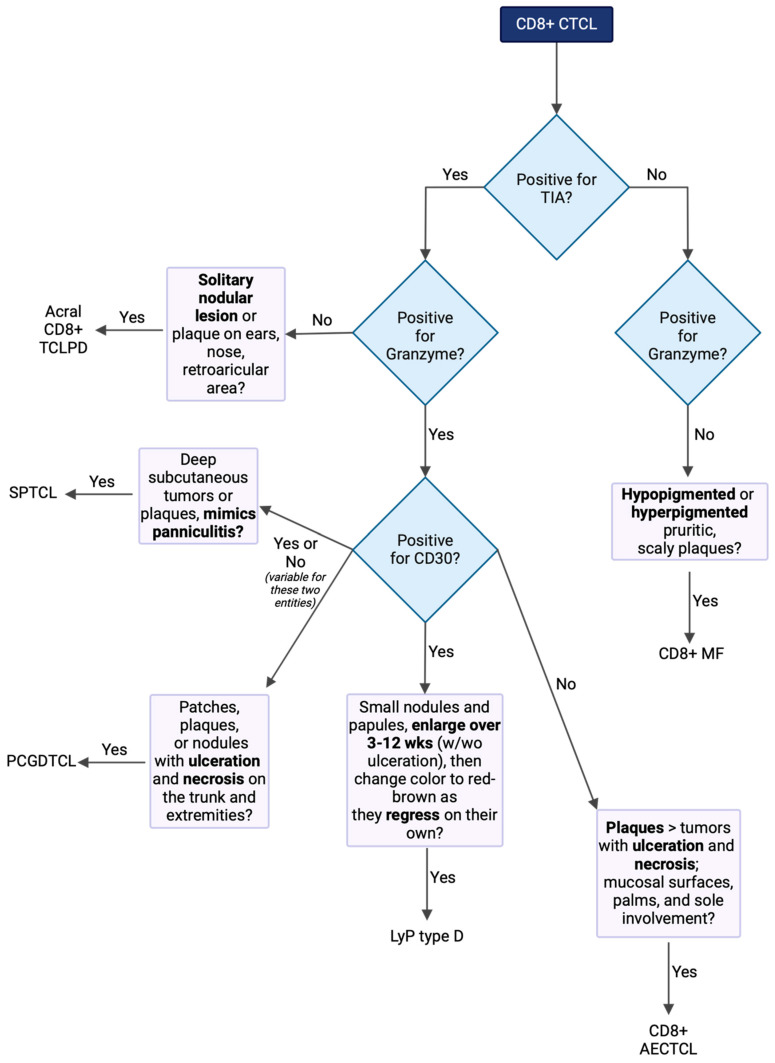
When constructing a diagnostic decision tree for CD8+ T-cell lymphoma, it is crucial to consider the key distinguishing features, particularly immunohistochemistry (IHC) differences and clinical decision points. The IHC differences, shown in the diamonds, include cytotoxic markers such as granzyme and TIA, as well as CD30 positivity. Clinical decision points, shown in rectangles, are also essential components of the decision-making process. Together, these elements help in accurately differentiating and diagnosing CD8+ T-cell lymphoma.

When diagnosing CD8+ CTCL, it can be beneficial to follow a logical algorithm that highlights the integration of pathology, immunohistochemistry (IHC), and clinical features (Figure 2). Key decision points to consider include T-cell differentiation markers such as CD4 and CD8 positivity, cytotoxic markers like granzyme and TIA, CD30 positivity, and the overall clinical presentation.

## 2. CD8+ Mycosis Fungoides (MF)

Mycosis fungoides (MF) is the most common of the CTCL subtypes, encompassing 65% of all CTCLs, the vast majority of which are CD4+, with an estimated less than 5% of cases of MF showing a CD8+ phenotype [3,6]. However, the true incidence of CD8+ MF may not be fully appreciated, as cases of clinical and histologic features showing classic MF changes may not be immunophenotyped. While CD8+ MF is most frequently observed in children presenting with hypopigmented lesions (often under the term “hypopigmented MF”), CD8+ MF typically presents in adults as patches and/or plaques that are often associated with pruritus or a burning sensation, but patients may be asymptomatic [6]. Histopathology most often shows features similar to CD4+ MF (albeit with the atypical T-cells showing a CD8+ phenotype), including an epidermotropic infiltrate, Pautrier microabscesses, and cerebriform nuclei [3]. Treatment varies depending on the stage of the disease, ranging from skin-directed therapies (e.g., topical steroids, topical retinoids, topical nitrogen mustard gel, phototherapy, and electron beam radiation therapy) to systemic therapies (e.g., oral bexarotene, interferons, extracorporeal photopheresis, pralatrexate, brentuximab, chemotherapy, or, in advanced or refractory cases, allogeneic stem cell transplantation).

A diagnosis of CD8+ MF, however, always raises some concern for early findings that may ultimately manifest as CD8+ AETCL. In this regard, reports of disease progression of CD8+ MF have been mixed, with some initially observing that it has a more aggressive nature than its CD4+ counterpart [7], while other cases note a more indolent course [6,8,9]. A recent molecular analysis of a case of hypopigmented MF showed a CD8+-dominant clone which had no malignant features compared to a reference population, suggesting that this case of hypopigmented MF may be a malignancy of CD4+ T-cells with the CD8+ population being reactive [10]. A close clinical follow-up with repeat biopsies of new or clinically different lesions is therefore warranted in patients with a diagnosis of CD8+ MF. Furthermore, when considering case three, it is interesting to note that atopic dermatitis-like Sézary syndrome has been represented through a small cohort of 13 patients to have a higher average age at diagnosis of 60.2 years and a diagnostic delay mean of 7.2 years [11]. However, prior work has demonstrated that the prevalence of atopy in a patient with mycosis fungoides and Sézary syndrome is approximately 11%, while the general population has a prevalence of 17–40% [12].

## 3. Lymphomatoid Papulosis (LyP), Type D

CD8+ lymphomatoid papulosis (LyP), also called LyP type D, is characterized by papulonodular lesions that erupt in clusters and self-resolve over 3–12 weeks. The number of lesions and degrees of growth, ulceration, and scarring can vary greatly between patients, which is why LyP is often considered on a spectrum of CD30+ LPDs with PCALCL [3]. A histopathologic assessment demonstrates an epidermotrophic CD8+ infiltrate that is often also CD30+ (and is typically also CD3+, CD4−, CD8+, CD25+, CD45RO+, and CD56+/−) [3]. Treatment options include topical steroids, ultraviolet light therapy, or low-dose methotrexate, with brentuximab being available for the more treatment-resistant CD30+ cases [3]. Although LyP generally carries a good prognosis, it is regularly associated (~45%) with various other malignancies [13]. Notably, patients with CD8+ LyP were less likely to develop a secondary lymphoma than CD8-negative types of LyP [14]. CD8+ LyP is differentiated from other CD8+ lymphoproliferative diseases by its clinical course, characterized by self-resolving papules and nodules, albeit often with scarring. CD8+ LyP is distinguished from CD8+ PCALCL in that the latter manifests as uni- or pauci-lesional tumors that are substantially less likely to show self-resolution. While CD8+ LyP may show epidermotropic cytotoxic T-cells in association with epidermal necrosis as in CD8+ AETCL, the more aggressive latter diagnosis will be fixed and grow as more rapidly advancing plaques and tumors.

## 4. Subcutaneous Panniculitis-like T-Cell Lymphoma (SPTCL)

Subcutaneous panniculitis-like T-cell lymphoma (SPTCL) is a rare CTCL representing 1% of all entities. It is characterized by deep subcutaneous tumors or plaques with overlying erythema most commonly seen on the trunk and extremities that mimics panniculitis [3]. There has also been a published case of SPTCL with indurated lesions without overlying erythema [15]. Immunophenotypically, SPTCL is most often CD4-, CD5-, CD8+, and CD56- and is positive for cytotoxic markers granzyme B and TIA-1 [3]. Histopathology classically demonstrates an infiltration of atypical alpha/beta T-cell lymphocytes and the subcutaneous tissue often rimming individual fat cells [15,16,17]. Systemic symptoms, otherwise known as B symptoms, are classically seen and frequently include malaise fatigue, fever, chills, and weight loss [3,16]. Furthermore, SPTCL is often complicated by hemophagocytic lymphohistiocytosis (HLH) [18]. While concurrent HLH may not portend a more rapidly progressive disease or worse prognosis, it may be associated with a lower survival rate in older patients [18]. The median age of onset is 36 years, with approximately one-fifth of patients being under the age of 20 when diagnosed [3,17]. This disease is two times more common in females than in males [3,17]. One out of five patients with SPTCL have an associated autoimmune disease, most commonly systemic lupus erythematosus, but Sjogren’s syndrome, type 1 diabetes mellitus, multiple sclerosis, and Reynaud’s disease were also found to be associated [3,19]. Even without treatment, SPTCL has an excellent prognosis, with a 5-year disease-specific survival rate of more than 85%. Treatment options include prednisone, methotrexate, bexarotene, romidepsin, and, in some cases, chemotherapy involving agents such as doxorubicin [3,19,20,21,22]. Interestingly, there have been two case reports published demonstrating a potential association between COVID-19 vaccination and the development of SPTCL [16,17].

## 5. Primary Cutaneous Gamma/Delta T-Cell Lymphoma (PCGDTL)

Although rare, at less than 1% of all CTCLs, primary cutaneous gamma delta T-cell lymphoma (PCGDTL) is highly aggressive and is frequently lethal as it is often refractory to treatment [3]. PCGDTL often presents as erythematous to violaceous patches, plaques, or nodules with ulceration or epidermal necrosis on the trunk and extremities [3,23]. PCGDTL occurs in the same frequency in both men and women, and the median age of patient presentation is in the range from the fourth to sixth decades of life [3,24,25]. Systemic B symptoms, including night sweats, weight loss, malaise, and fever, are seen in patients with PCGDTL [3]. Mucosal and extra-nodal site involvement is common, but the involvement of the spleen lymph nodes or bone marrow is rare for this subtype. The medium survival time has been reported to be within the range of 15–31 months [3,25]. There is no standardized treatment regimen for this rare disorder. The most utilized treatment reported was anthracycline-based chemotherapy, though most patients experienced relapse [24]. Poor prognostic factors include subcutaneous involvement and ulceration, CD56 positivity, CD95 positivity without CD8 positivity, central nervous system involvement, hemophagocytic lymphohistiocytosis syndrome, and an age greater than 40 years [24]. Immunophenotypically, PCGDTL is typically positive for CD2, CD3, and CD56, as well as producing cytotoxic enzymes TIA-1 and granzyme B; negative for CD4 and CD5; and variable for CD8 [3,24]. Some published cases have been double negative for CD4 and CD8 [26]. As indicated by the entity name, they demonstrate TCR gamma/delta gene rearrangement, and IHC utilizing antibodies against TCR gamma and delta can help confirm the disease [3,23]. SPTCL and PCGDTCL were previously grouped under the same subheading of SPTCL since they have a similar histopathology of a lobular panniculitis-like morphology with a cytotoxic T-cell infiltrate [3]. However, due to their different prognoses, with SPTCL having an excellent prognosis and PCGDTCL being nearly always fatal, they are now considered different entities [3].

## 6. CD8+ Aggressive Epidermotropic Cutaneous T-Cell Lymphoma (AECTCL)

The most aggressive of the CD8+ CTCLs, CD8+ aggressive epidermotropic cutaneous T-cell lymphoma (AECTCL) is characterized by a clinical presentation of ulcerated, often necrotic plaques and/or tumors which may involve mucosal surfaces and the palms and soles [3]. Biopsies demonstrate a dense CD8+ epidermotrophic infiltrate with irregular hyperchromatic nuclei and epidermal (or in tumors, more extensive dermal) necrosis [3]. Although CD8+AECTCL is rare, comprising less than 1% of TCLs, its potential for rapid progression is associated with a poor 5-year mean survival of ~18% (median survival time of 23–32 months) [3]. Additionally, treatment is challenging as there are no randomized control trials due to its infrequency, and these patients are often excluded from clinical trials with new agents due to overall poor prognosis and rapid progression [3]. Initial management options usually include multi-agent systemic chemotherapy and/or radiation therapy to involved lesions [27]. Interestingly, treatment with interferon alpha has been demonstrated to worsen the disease and should subsequently be avoided in patients with this diagnosis [28]. Therefore, when suspected, it is important to start treatment early and to consider allogeneic stem cell transplantation in appropriate patients if the disease is chemoresponsive. It may also be prudent to conduct next-generation sequencing (NGS) in cases of CD8+ AETCL to explore potential matches with alternative therapeutic agents, thereby enhancing personalized treatment strategies.

## 7. Acral CD8+ T-Cell Lymphoproliferative Disorder (Acral CD8+ TCLPD)

The indolent acral CD8+_T-cell lymphoproliferative disorder (acral CD8+ TCLPD) most frequently presents as a nodular solitary lesion on the face, nose, and/or ear but can also less often present on the hands, arms, legs, or feet [4]. Acral CD8+ TCLPD is rare, representing less than 1% of all CTCLs, and has a significantly less aggressive course than other CTCL subtypes (with the median 5-year-survival ranging between 75 and 100%), and therefore typically requires less intensive therapies [29]. Histopathology demonstrates dermal diffuse infiltrate with a nodular pattern involving the subcutis and an absence of epidermotropism [4]. Lesions are typically responsive to treatment with spot radiotherapy or surgical excision, and regression after treatment can occur in approximately 20–45% of patients [4,29]. When suspected, it is important to counsel patients on the indolent nature in contrast to other CD8+ CTCL counterparts and to ensure patients are not overtreated.

## 8. Other Diagnostic Considerations: Peripheral T-Cell Lymphomas Not Otherwise Specified (PTCL-NOS) and Natural Killer T-Cell (NK/T) Lymphoma and Lymphoproliferative Disorders

When diagnosing CTCL variants, including those with a CD8+ phenotype, it is important to consider several diagnoses of exclusion after ruling out the more common diagnoses mentioned earlier. Peripheral T-cell lymphoma not otherwise specified (PTCL-NOS) is a defined entity of peripheral T-cell lymphomas that does not fit within other defined entities [30]. For this reason, PTCL-NOS should be considered in patients where cutaneous T-cell lymphoma can still be expected despite the lack of perfect existence of all the histopathologic and clinical criteria for one of the aforementioned entities. There are a wide range of clinical presentations for cutaneous manifestations of PTCL-NOS; for example, one published case demonstrated a PTCL-NOS mimicking periorbital cellulitis, while another case report showed a presentation similar to eczematous dermatitis or graft-versus-host disease in a post-transplant pediatric patient [31,32]. Additionally, a subtype of PTCL-NOS includes natural killer T-cell lymphoproliferative disorders, which can involve the skin, especially the extra-nodal nasal type (ENKTL) as well as the hydroa vaccinforme-like lymphoproliferative disorder (HVLLPD) type [33]. ENKTL typically presents as nodular, abscess-like lesions most commonly affecting the nasopharynx, while HVLLPD typically presents as papulovesicular lesions with ulceration in photo-exposed areas [33,34]. Immunophenotypically, natural killer T-cell lymphoproliferative disorders are often double negative for CD4 and CD8, though they can be CD8-positive, especially the HVLLPD subtype [33,34]. Both subtypes are classically Epstein–Barr virus-positive, which can aid in diagnosis [33].

## 9. Conclusions

Clinical or histologic findings of lymphomatous plaques and/or tumors with necrosis often prompt IHC staining to help distinguish cutaneous lymphoma subtypes. Despite sharing CD8+ marker expression, the various entities of CD8+ MF, LyP type D, SPTCL, PCGDTL, CD8+ AECTCL, and acral CD8+ TCLPD demonstrate different clinical features, have a variety of different prognoses, and all have characteristic treatment regimens. Due to the rarer nature of these diseases, the absence of a fuller immunophenotypical picture, and a lack of genetic data to classify these subtypes, it is imperative to integrate clinical, histologic, and immunohistochemical findings to determine an accurate diagnosis and appropriate treatment plan (Table 2).

In summary, we discuss variants of CD8+ cutaneous lymphomas comprising a spectrum of disease subtypes. Although they may all show a CD8+ phenotype, the various entities CD8+ MF, LyP type D, SPTCL, PCGDTL, CD8+ AECTCL, and acral CD8+ TCLPD have distinctive clinical features, variable prognoses, and recommended treatment regimens. These variants highlight the importance of integrating clinical presentation and lesions’ morphology with histopathologic, immunophenotypic, and molecular features to determine the accurate diagnosis, prognosis, and treatment plan for each patient.

## Figures and Tables

**Table 1:** Frequency, 5-year prognosis, and immunohistochemistry findings for each classified entity of CTCL. Data compiled from various tables in Atlas of Cutaneous Lymphomas: Classification and Differential Diagnosis [3], Kempf et al. [4], and Guitart et al. [5].

Entity Name	Frequency (% of T-Cell Lymphomas)	5-Year Prognosis (%)	Immunohistochemistry Findings					
			CD3	CD4	CD8	CD30	CD56	Other
MF	65%	75–98%, can vary by subtype	+	+	- (rarely +)	-	-	CD25-
SS	4%	10–33%	+	+	-	-	-	CD26-, CD27+
LyP: Type A,C	16%	100%	+	+	-	+	-	ALK-, CD15-
LyP: Type B			+	+	-	-	-	ALK-
LyP: Type D			+	-	+	+	-	ALK-, βF1+
PCALCL	10%	90%	+	+	-	+	-	ALK-
SPTCL	1%	85–90%	+	-	+	±	-	βF1+
PCGDTL	<1%	N/A, median survival is 15 months	+	-	±	±	+	Beta F1-, TCRγ+
CD8+ AECTCL	<1%	18%	+	-	+ (rarely -)	-	-	CD45RA- or partially +, βF1+
Acral CD8+ TCLPD	<1%	75–100%	+	-	+	-	-	
CD4+ SMCLPD	<1%	>90%	+	+	-	-	-	

MF: mycosis fungoides, SS: Sézary syndrome, LyP: lymphomatoid papulosis, PCALCL: primary cutaneous anaplastic large cell lymphoma, SPTCL: subcutaneous panniculitis-like T-cell lymphoma, PCGDTL: primary cutaneous gamma/delta T-cell lymphoma, CD8+ AECTCL: CD8+ aggressive epidermotropic cytotoxic T-cell lymphoma, acral CD8+ TCLPD: CD8+ acral T-cell lymphoproliferative disorder, CD4+ SMCLPD: CD4+ small or medium T-cell lymphoproliferative disorder.

**Table 2:** Summary of clinical course, histopathologic findings, and typical treatment and prognosis for each entity of CTCL described in our cases. Information obtained from Atlas of Cutaneous Lymphomas: Classification and Differential Diagnosis [3], Kempf et al. [4], Guitart et al. [5], and Dermatopathology, Cutaneous Lymphomas [35].

CD8+ Subtype	Clinical Features	IHC	Treatment
CD8+ MF	**Hypopigmented** or **hyperpigmented** pruritic, scaly plaques	CD3+, CD7− CD8+, CD30+/− CD56−, CCR4+ **Cytotoxic granules-** **(TIA-GZM-)**	Early: topical steroids and/or retinoids, phototherapy, RT Advanced: bexarotene, IFNα, ECP, chemotherapy, stem cell transplant
LyP type D	Small nodules and papules, **enlarge over 3–12 wks** (w/wo ulceration), then change color to red-brown as they **regress** on their own	CD3+, CD4− CD8+, CD25+ **CD30+**, CD45RO+ CD56+/− **Cytotoxic granules+ (TIA+GZM+)**	Topical steroids, phototherapy, low-dose MTX **Recurrence is common**
SPTCL	Deep subcutaneous tumors or plaques, **mimics panniculitis**	CD4−, CD5−, CD8+, CD30+/−, and CD56− **Cytotoxic granules+ (TIA+GZM+)**	Prednisone, MTX, bexarotene, romidepsin, chemotherapy
PCGDTL	Erythematous to violaceous patches, plaques, or nodules with **ulceration** and **necrosis** on trunk and extremities	CD2+, CD3+, CD4−, CD5−, CD8 +/−, CD30+/−, CD56+, **Cytotoxic granules+ (TIA+GZM+)**	Chemotherapy, **though almost always fatal**
CD8+ AECTCL	**Plaques** > tumors with **ulceration** and **necrosis**; mucosal surfaces, palms, and sole involvement	CD3+, CD4− CD5−, CD8+ CD30− **Cytotoxic granules+ (TIA+GZM+)**	Chemotherapy, localized RT **IFNα may worsen disease**
Acral CD8+ TCLPD	**Solitary nodular lesion** or plaque on ears, nose, or retroarticular area	CD3+, CD4− CD8+, CD30− CD56− **Cytotoxic granules+/−** **(TIA+, GZM−)**	RT or surgical excision

RT: radiation therapy; IFNα: interferon alpha; MTX: methotrexate.

## Abbreviations

CD	cluster of differentiation
CD4+ SMCLPD	CD4-positive small or medium T-cell lymphoproliferative disorder
CD8+ AECTCL	CD8-positive aggressive epidermotropic cytotoxic T-cell lymphoma
acral CD8+ TCLPD	CD8-positive acral T-cell lymphoproliferative disorder
CTCL	cutaneous T-cell lymphoma
FDG	F-18 fluorodeoxyglucose
IHC	immunohistochemistry
LyP	lymphomatoid papulosis
MF	mycosis fungoides
PCALCL	primary cutaneous anaplastic large cell lymphoma
PCGDTL	primary cutaneous gamma/delta T-cell lymphoma
PET-CT	positron emission tomography computed tomography
SPTCL	subcutaneous panniculitis-like T-cell lymphoma
SS	Sézary syndrome
TCL	T-cell lymphoma
